# Evaluation of ingesting Dyma-Burn Xtreme, a thermogenic dietary supplement, on hemodynamic and ECG responses in healthy, young males and females

**DOI:** 10.1186/1550-2783-9-S1-P12

**Published:** 2012-11-19

**Authors:** Craig Jones, Sara Hayward, Stacie Urbina, Cliffa Foster, Shawn Wells, Rob Wildman, Bill Campbell, Lem Taylor, Colin Wilborn

**Affiliations:** 1 University of Mary Hardin-Baylor, Human Performance Lab, Belton, TX 76513, USA; 2 Dymatize Nutrition, Farmers Branch, TX 75234, USA; 3 University of South Florida, Exercise & Performance Nutrition Lab, Tampa, FL 33544, USA

## Background

Ingestion of caffeine is traditionally thought to acutely elevate both blood pressure and heart rate based on the stimulatory properties that it exerts on both the central and peripheral nervous systems, and this effect is primarily dependent on the dose as well as an individual’s sensitivity to caffeine. The purpose of this study was to evaluate the safety of the ingestion of a proprietary thermogenic dietary supplement, including the ingredients caffeine, green tea extract, raspberry ketones, and L-Carnitine on ECG and hemodynamic responses.

## Methods

In a double-blind, crossover design 6 male (N = 6, 23.6 ± 5.8 years, 180.5 ± 6.0 cm, 89.7 ± 7.1 kg, 16.5 ± 7.1 %BF) and 6 female (N = 6, 21.3 ± 3.8 years, 162.0 ± 6.0 cm, 64.1 ± 7.4 kg, 28.8 ± 7.6 %BF) moderate caffeine users (< 200 mg/day) reported to the lab on a 12 hour fast and had a baseline heart rate (HR), blood pressure (SBP and DBP), and ECG variables (RR interval, PR interval, QRS duration, and QT interval) were assessed. Subjects consumed either a 2 capsule serving of Dyma-Burn Xtreme (DBX) or placebo (PLC) and had HR, SBP/DBP assessed at the end of each hour; and assessed ECG variables in a supine position at 1 hour (1HR), 2 hour (2HR), 3 hour (3HR), and 4 hour (4HR) post consumption. All data was analyzed utilizing a 2x5 ANOVA and one-way ANOVAs were used in the case of a significant interaction. A significance value of 0.05 was adopted throughout.

## Results

No significant (p < 0.05) time or group x time interaction effects were observed for SBP, DBP, and HR. SBP delta responses (DBX vs. PLC) from baseline are as followed: 1HR (12.4 ± 11.8 vs. 1.75 ± 10.4 mmHg), 2HR (10.0 ± 14.0 vs. 0.0 ± 7.9 mmHg), 3HR (13.5 ± 22.4 vs. -2.5 ± 8.1 mmHg), and 4HR (8.3 ± 10.5 vs. 1.5 ± 10.6 mmHg). Delta responses from baseline for DBP are as followed (DBX vs. PLC): 1HR (4.8 ± 7.4 vs. 0.6 ± 7.9 mmHg), 2HR (-0.25 ± 13.2 vs. -1.0 ± 7.2 mmHg), 3HR (6.7 ± 20.9 vs. -4.5 ± 10.1 mmHg), and 4HR (1.25 ± 6.8 vs. 1.1 ± 11.0 mmHg). The observed delta responses for HR are as followed (DBX vs. PLC): 1HR (-3.0 ± 6.2 vs. -2.5 ± 5.5 bpm), 2HR (-2.9 ± 6.5 vs. -1.0 ± 10.0 bpm), 3HR (-2.3 ± 5.6 vs. -0.5 ± 8.7 bpm), and 4HR (-1.4 ± 6.8 vs. -0.3 ± 7.4 bpm). No significant (p < 0.05) group or time differences were observed for ECG intervals (RR, PR, and QT) and QRS duration. Additionally, no observed changes in ECG rate and rhythm abnormalities (i.e., PVCs, arrhythmias, etc.) were seen across any time points.

## Conclusion

Acute ingestion of DBX had no significant effects on hemodynamic function and various ECG intervals over the four-hour observation period in daily caffeine users. The stimulatory effects that traditionally occur following caffeine ingestion was not observed, which could be explained by a decreased sensitivity to caffeine from regular consumption.

